# Tissue‐Equivalents of Lymphoid Clonal Hematopoiesis of Indeterminate Potential (L‐CHIP) and Germline‐Derived Lymphoproliferations: Possible Caveats for Hematopathologists

**DOI:** 10.1002/hon.70145

**Published:** 2025-10-11

**Authors:** Magdalena M. Brune, Ivana Bratic Hench, Stefan Dirnhofer, Alexandar Tzankov

**Affiliations:** ^1^ Institute of Medical Genetics and Pathology University Hospital Basel Basel Switzerland

**Keywords:** clonal hematopoiesis, germline‐derived variant, lymphomagenesis, lymphoproliferation, next generation sequencing

## Abstract

Clonal hematopoiesis of indeterminate potential (CHIP) is a predisposing condition to lymphoma development. CHIP carrying mutations that are recurrently found in lymphomas are designated as L‐CHIP. We presume that bone marrow‐derived L‐CHIP populations are able to expand and manifest in peripheral lymphoid tissues, where they could hence be called *L‐CHIP tissue‐equivalents*. There, they may proliferate and foster unexplained follicular hyperplasias, and, thus, potentially represent an early precursor of lymphoma. Analogously, we hypothesize that certain germline‐derived mutations lead to lymphoproliferations (*germline‐derived lymphoproliferations*) in otherwise healthy individuals. We collected seven exceptional cases of symptomatic nodal and extranodal lymphoid hyperplasias, which were all morphologically suspicious and displayed somatic and/or germline‐derived mutations recurrently found in B‐cell lymphomas. One patient developed follicular lymphoma after 8 years carrying the same non‐productive immunoglobulin rearrangement detected in the initial biopsy. L‐CHIP tissue‐equivalents and germline‐derived lymphoproliferations potentially represent first steps in lymphomagenesis and knowledge about their existence might be of diagnostic utility in challenging cases of (atypical) lymphoproliferations. With histology, immunohistochemistry, and molecular testing, such lesions can be identified in situ.

## 1 Introduction

In many solid neoplasms, invasive cancer is preceded by pre‐malignant lesions acquiring sequential molecular alterations over time. Due to the circulatory capacity of lymphocytes, this notion of precursor lesions is thought to be applicable to lymphomas only to a very limited extent. One recognized precursor state of hematologic neoplasms is the age‐related phenomenon of clonal hematopoiesis of indeterminate potential (CHIP). It involves clonal expansion of hematopoietic populations that have acquired at least one somatic mutation. In 2021, Niroula et al. sub‐classified CHIP into myeloid (M‐CHIP) and lymphoid (L‐CHIP), depending on the underlying mutation(s) [1]. Although M‐CHIP has been described mostly in the myeloid context, recent studies and case reports suggest that M‐CHIP can give rise to lymphoid neoplasms after the acquisition of additional genetic alterations [2, 3, 4, 5]. Concurrently, van Beck and colleagues reviewed on L‐CHIP as a predisposing condition to lymphomas and concluded that its detection might provide an important opportunity to identify high‐risk patients [6]. Albeit L‐CHIP is not an uncommon condition among elderly individuals, and by far not every L‐CHIP carrier will develop a lymphoma [1]. For this reason, more precise instruments must be found to dissect high‐risk CHIP carriers from the residual population, thereby minimizing the numbers needed to screen. Another lymphoma predisposing condition is the presence of certain germline variants in patients with inborn errors of immunity, such as autoimmune lymphoproliferative syndrome (ALPS), ataxia‐teleangiectasia (A‐T), immunodeficiency caused by germline loss‐of‐function mutations in *STAT3* etc. [7, 8, 9, 10, 11]. Apart from that context, little is known about the impact of germline‐derived variants on the individual risk of lymphomagenesis.

We hypothesize that bone marrow‐derived L‐CHIP populations expand and populate peripheral nodal and extranodal lymphoid tissues, where they proliferate and could hence be called *L‐CHIP tissue‐equivalents*. There, they may disturb and circumvent the germinal center selection machinery, thus, providing fertile ground for the expansion of otherwise apoptosis‐prone defective B‐cells, and thereby fostering autonomous lymphoproliferations. In analogy, we assume that certain germline‐derived variants lead to the development of atypical lymphoproliferations in otherwise healthy individuals. Such lymphoproliferations may state the earliest detectable precursor lesions of lymphomas. In our opinion, they can be identified in situ by histological, immunohistochemical, and molecular testing based on next generation sequencing (NGS) for analysis of mutational profiles and B‐cell receptor (BCR) clonality.

## 2 Case Presentation

Our hypothesis is based on observations in research and during routine histopathological work‐up of several exceptional cases (summarized in Table 1). Recently, our group investigated the usefulness of immunohistochemical assessment of H3K27m3 (tri‐methylated histone H3 at position lysine 27) expression in the differential diagnosis between follicular lymphoma (FL) and reactive follicular hyperplasia (FH) [12]. Overexpression of H3K27m3 in the setting of FH served as a caveat, as it potentially represents a read‐out of dysfunctional methylation in the germinal centers, which is characteristic of lymphoid neoplasms [12]. We identified cases of FH that were clinically symptomatic, morphologically suspicious and—upon further molecular analysis—revealed variants in genes recurrently altered in B‐cell lymphomas. Molecular analysis was performed by using a custom‐designed IonTorrent AmpliSeq NGS lymphoma panel (Thermo Fisher Scientific, Carlsbad, CA, USA), as described previously [13, 14, 15]. Details on the applied immunohistochemistry, tissue preparation, sequencing conditions, quality control, and variant annotation can be found in the Supporting Information S1 and Tables S2 and S3. The study was approved by the Ethics Committee for Northwestern and Central Switzerland, approval number 2023‐00907.

**TABLE 1:** Overview of the reported cases, including molecular details of next generation sequencing (NGS).

Case ID, (age, Sex)	Location	Histology	H3K27m3 expression in GC	PTEN expression in GC	NGS result	B‐cell clonality (% of the dominant clone)	Germline testing (tested tissue)	Follow‐up
1 (30, f)	Cervical LN	Follicular and paracortical hyperplasia	Over‐expression	Unremarkable	***KMT2D*** (somatic, VUS): c.1445 C > A (p.A482 E), VAF 7%; c.1448 T > C (p.L483S), VAF 6% ***KMTD2*** (germline, VUS) c.7400 A > C (p.H2467P), VAF 43%	Polyclonal	***KMTD2:*** c.7400 A > C (p.H2467P), VAF 43% (Lymphocyte‐depleted adjacent connective tissue)	Within a follow‐up period of 9 years still alive and without evidence of lymphoma
2 (68, m)	Inguinal LN	Focal atypical follicular lympho‐proliferation	Over‐expression	Loss of expression	***B2M*** (somatic, likely pathogenic): c.67 + 1G > C, VAF 6%	Non‐productive, monoclonal rearrangement IGKV2‐28 ‐ IGKdel (34%)	No mutation found (lymphocyte‐depleted adjacent connective tissue)	Follicular lymphoma G3A 8 years later with: ***SOCS1*:** p.I182S (c.545 T > G), VAF 22% ***KMT2D*:** p.Q1178* (c.3532 C > T), VAF 8%, same monoclonal rearrangement IGKV2‐28 ‐ IGKdel
3 (7, f)	Tonsil	Atypical germinal center hyperplasia	Unremarkable	Unremarkable	***ATM*** (germline, pathogenic): c.6095 G > A (p.R2032 K), VAF 59%	Polyclonal	***ATM:*** c.6095 G > A (p.R2032 K), VAF 50% (Lymphocyte‐depleted adjacent connective tissue)	Within a follow‐up period of 6 years still alive and without evidence of lymphoma
4 (64, m)	Thyroid	Atypical follicular lympho‐proliferation	Unremarkable	Unremarkable	***ATM* (**germline, VUS): c.4709 T > C (p.V1570 A), VAF 42%	*Left thyroid:* Non‐productive, biclonal rearrangement IGKV2‐28 ‐ IGKJ1 (22%) & IGKCint—IGKdel (21%) *Right thyroid:* Non‐productive, monoclonal rearrangement IGKV2‐28 ‐ IGKJ1 (17%)	***ATM:*** c.4709 T > C (p.V1570 A), VAF 53% (Tumor remote thyroid and connective tissue)	No data
5 (79, f)	Lung	Interstitial follicular hyperplasia	Not performed	Loss of expression	***PTEN*** (germline, pathogenic): c.834 C > G (p.F278 L), VAF 18%	Polyclonal	***PTEN***: c.834 C > G (p.F278 L), VAF 14% (Thyroid tissue)	Within a follow‐up period of 12 years still alive and without evidence of lymphoma
6 (53, f)	Conjunctiva	Follicular conjunctivitis	Not performed	Loss of expression	***PTEN*** (pathogenic): c.834 C > G (p.F278 L), VAF 19%	Polyclonal	Not performed	Within a follow‐up period of 8 years still alive and without evidence of lymphoma
7 (57, m)	Perirenal soft tissue	Atypical follicular lympho‐proliferation	Unremarkable	Unremarkable	***PRDM1*** (probably germline, unknown): c.2110 G > T (p.V704 F), VAF 50%	Polyclonal	Not performed	Within a follow‐up period of 3 years still alive and without evidence of lymphoma

Abbreviations: *ATM*, ataxia‐telangiectasia mutated; B2M, beta 2 microglobulin; f, female; H3K27m3, tri‐methylated histone 3 position lysine 27; IG, immunoglobulin; *KMT2D*, histone‐lysine N‐methyltransferase 2D; LN, lymph node; m, male; *PRDM1*, PR domain zinc finger protein 1; *PTEN*, hosphatase and tensin homolog; *SOCS1*, suppressor of cytokine signaling 1; VAF, variant allelic frequency.

**FIGURE 1:**
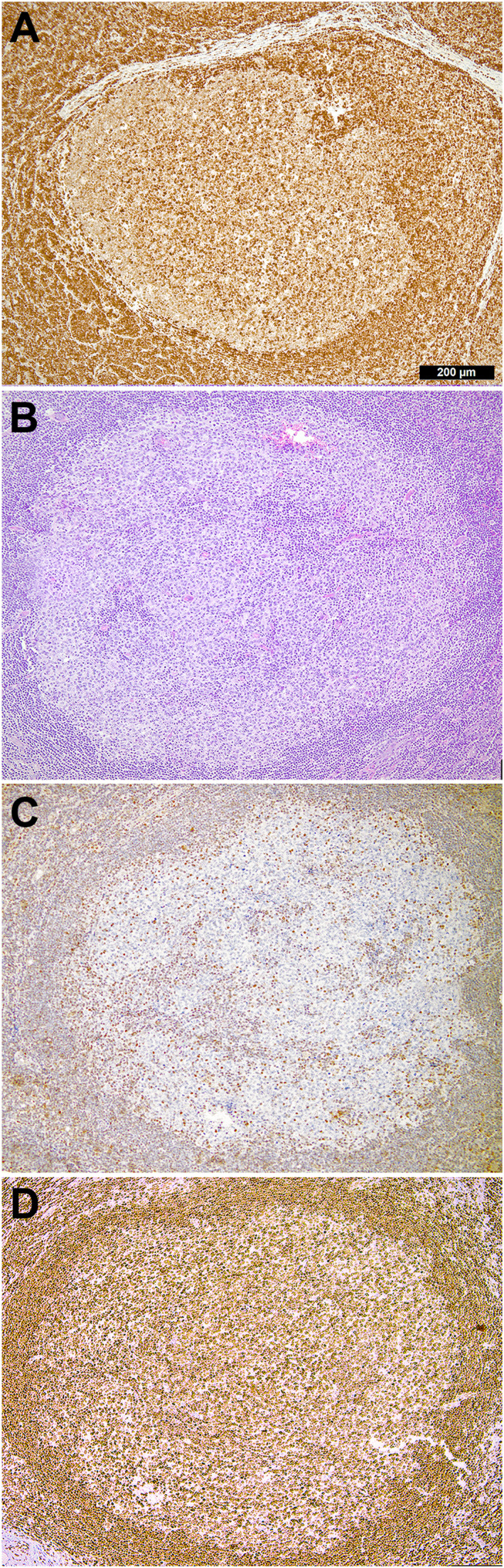
(A) Case 1: Abnormal immunohistochemical overexpression of H3K27m3 in an otherwise unremarkable follicular hyperplasia (FH) of a patient with three *KMT2D* variants of uncertain significance, of which one is germline‐derived; immunoperoxidase staining with the Cell Signaling cs9733 antibody. (B) Case 2: Conventional morphology of an abnormal (BCL2‐negative; not shown) germinal center (GC) rendered as atypical FH; H&E staining. (C) Case 2: Lost expression of PTEN in the respective GC; note the surrounding positively staining cells of the marginal zone; immunoperoxidase staining with the Cell Signaling 138G6 antibody. (D) Case 2: Abnormal immunohistochemical overexpression of H3K27m3.

Case 1 a 30‐year‐old female patient presented with massive FH of a cervical lymph node and H3K27m3 overexpression in the germinal centers (Figure 1A) [12]. Despite these morphological conspicuities, in the lymphoid tissue neither BCR clonality nor *EZH2* mutations nor *BCL2* rearrangements were detected. Instead, molecular evaluation with our accredited customized lymphoma NGS panel [15] revealed three *KMT2D* variants of uncertain significance (VUS), with variant allelic frequencies (VAF) of 6%, 7%, and 43%. Further investigation confirmed the germline origin of the variant with the highest VAF. We assume that the presence of this germline variant interfered the germinal center machinery, as proven by H3K27m3 overexpression. H3K27m3 overexpression in FL by far extends the proportion of *EZH2* mutant cases and it is postulated that the tri‐methylation of H3 is influenced by many more players than mutated *EZH2* alone and represents one of the final stretches of the disturbed epigenetic machinery in B‐cell lymphomagenesis, which also includes *KMT2D* [12, 16]. Additionally, in this case, the germline *KMT2D* variant might have facilitated the development of the two other (somatic) mutations in the same gene in a yet unknown way. It is also possible that the germline *KMT2D* variant promoted the emergence of the somatic variants already at the bone marrow‐level, creating germline‐derived L‐CHIP. This finding is in line with Niroula and colleagues [1], who described *KMT2D* among the most frequently altered genes in L‐CHIP.

Case 2 a 68‐year‐old male patient presented with an enlarged inguinal lymph node revealing a small architecturally disturbed area with H3K27m3 overexpression and loss of PTEN (Figure 1B–D). No *BCL2* rearrangements were detectable. At the molecular level, a likely pathogenic *beta‐2‐microglobulin* (*B2M*) splice‐site mutation with a VAF of 6% was detected, as well as a non‐productive IGK rearrangement (clone frequency 34%). Eight years later, the patient was diagnosed with a FL, grade 3A, with the same non‐productive IGK rearrangement, but without harboring the previously identified *B2M* variant. Instead, mutations in *SOCS1* (VAF 22%) and *KMT2D* (VAF 8%) were detected. These findings allow the assumption that the initial *B2M*‐mutated clone in the lymph node did not represent a direct precursor of the later FL, but a progeny that supported the germinal center survival of an erroneous non‐productive IGK‐rearranged B‐cell clone. Later, the constellation of the non‐productive/dysfunctional IGK rearrangement and additionally acquired mutations of the tumor suppressors *SOCS1* and *KMT2D* provided fertile ground for lymphomagenesis. Despite the loss of PTEN expression, no *PTEN* mutation was detected. The PTEN loss in this case can be explained by underlying epigenetic disturbances that might have led to an altered PTEN protein expression or a possible *PTEN* deletion may not have been accurately reflected by the applied technology.

Case 3 a 7‐year‐old girl child was diagnosed with a massive atypical germinal center reaction in the tonsil, being phenotypically inconspicuous for BCL2, PTEN, and H3K27m3 expression. NGS detected a pathogenic *ATM* variant (VAF 59%). Its germline derivation was proven and was in accordance with the patient's age, the characteristic VAF of 59% and its documented occurrence in A‐T (https://www.ncbi.nlm.nih.gov/clinvar/RCV000167946.14/) and. In this patient, this genetic alteration led to the clinically apparent unilateral enlargement of the tonsil with an atypical lymphoproliferation, but yet without other clinical signs of A‐T. In accordance, *ATM* mutations are characteristic of L‐CHIP [1].

Case 4 a 64‐year‐old male patient presented with a mass‐causing atypical follicular lymphoproliferation of the thyroid. Criteria for malignancy were not fulfilled (no BCL2 expression in the germinal centers, no clonal IGH rearrangement on multiplex PCR as well as no pathogenic mutation on NGS). Interestingly, NGS revealed the presence of an *ATM* VUS (VAF 42%) that, again, was germline‐derived. Furthermore, NGS detected a non‐productive IGK rearrangement [clone frequency 22% (left thyroid) and 17% (right thyroid)]. We hypothesize that the *ATM* mutation likely disturbed the germinal center reaction and with that the negative selection of an otherwise apoptosis‐prone ‐ because non‐productive ‐ IGK clone. Consequently, this clone most likely proliferated and led to the clinically detectable lymphoproliferation with irregularly formed germinal centers and marginalization of the thyroid tissue.

Cases 5&6 two 79 and 53‐year‐old female patients presented with unusual FH of the lung and follicular conjunctivitis, respectively [13, 14]. PTEN loss was observed in both cases by immunohistochemistry in the lymphoid cells within the germinal centers. Indeed, both cases displayed the same *PTEN* variant with VAF of 18% and VAF 19%, correspondingly. *PTEN* is a well‐known tumor suppressor gene, which is frequently altered in lymphoid malignancies and solid tumors [17]. As *PTEN* plays an important role in the germinal center reaction, *PTEN*‐altered L‐CHIPs could show a much higher tropism toward secondary lymphoid tissues than to the blood, not being among the commonly noticed L‐CHIP mutations [1]. In the lung case, sequencing of normal thyroid tissue revealed germline origin of the *PTEN* mutation, despite its low VAF. This, repeatedly, underlines the potential correlation between the existence of germline‐derived variants and the presence or evolution of (atypical) lymphoproliferations. Due to limited tissue availability, germline testing could not be performed in the second patient.

Case 7 a 57‐year‐old male patient displayed a tumorous atypical follicular lymphoproliferation of the perirenal soft tissue that led to nephrectomy for radiologically suspected malignancy. Histologically, the differential diagnosis of a FL was rendered but could not be proven with immunohistochemistry. No clonal BCR rearrangement on multiplex PCR was found as well as any pathogenic variant on NGS. Merely, a *PRDM1* VUS was identified, which could be associated with the occurrence of the lymphoproliferation. Because of the VAF of 50%, a germline derivation is assumed. *PRDM1,* which is described as one of the most frequently mutated genes in marginal zone lymphomas, acts as a tumor suppressor gene and is located on a locus frequently deleted in lymphoid tumors [18, 19].

## 3 Discussion

The above‐described cases support the concept of detectable L‐CHIP tissue‐equivalents and germline‐driven nodal and extranodal (autonomous) lymphoproliferations. Importantly, all these cases were clinically symptomatic and in need of surgical intervention at least for diagnostics. They were morphologically and/or phenotypically conspicuous or showed unexpected clinical presentations such as extranodal locations, exuberant or asymmetrical findings. Still, our assumptions are based on a limited collection of cases and include many theoretical considerations that have not yet been proven in a larger cohort. Thus, whether the presence of this type of lymphoproliferations is associated with a higher risk of lymphoma development cannot be answered with this limited case collection. Nevertheless, our observations are in line with the known role of certain germline mutations in patients with inborn errors of immunity [7, 8, 9, 10, 11], are supported by the findings made by Niroula et al. [1] and fit well with the von Beck et al.’s model of *central* and *peripheral* L‐CHIP where, depending on the timepoint of the mutation occurrence during lymphocyte development, either IG receptor‐diverse (*central*) or receptor‐homogenous (*peripheral*) progenies are produced [6]. These deliberations are particularly important in the context of Cases 2 and 4, in which non‐productive IG rearrangements were present. Case 2 might indeed represent a *peripheral* L‐CHIP, producing receptor‐homogenous progenies that later on potentially gave rise to the FL. Case 4 might represent a germline‐derived equivalent, in which the *ATM* variant impaired the germinal center machinery, possibly facilitating the survival of a non‐productive IGK clone and inducing the clinically apparent lymphoproliferation within the thyroid.

Currently, the presence of high frequency t(14;18)‐bearing clones in healthy individuals is regarded as the first predictive biomarker of an increased risk for FL and the WHO recognizes in situ follicular B‐cell neoplasm (ISFN) as the earliest histologically detectable tissue involvement by circulating t(14;18) positive cells [20]. Of note, there is no concept for “BCL2‐negative ISFN”, although 15% of FL do not harbor *BCL2* rearrangements. All our cases that could be tested were BCL2‐negative and it seems possible that the encountered lesions might represent early precursors of *BCL2*‐independent lesions that have not been put into context yet.

For sure, there is further need to clarify the role of L‐CHIP tissue‐equivalents, their origin, and their potential status as a precursor lesion, also taking into account the significance of germline‐derived variants in individuals without apparent errors of immunity. Therefore, our considerations should be substantiated within a systematic approach in larger cohorts with long‐term follow‐up, also considering bone marrow and peripheral blood findings. For now, existence of such lesions should be kept in mind by hematopathologists when dealing with atypical lymphoproliferations that do not fulfill diagnostic criteria for lymphoma but are still suspicious.

In summary, our data provide first hints for the existence of L‐CHIP tissue‐equivalents somewhat analogous to lymphoproliferations associated with germline mutations. Although the clinical significance of these lymphoproliferations is still unclear, knowledge of their existence may help for proper interpretation and avoidance of mis‐ and especially overdiagnosis, but also alert the responsible clinician to a possible, yet unproven, increased risk of developing lymphoma in the affected patients.

## Author Contributions

All authors had final approval of the submitted version. M.M.B. conceptualization, data curation, formal analysis, investigation, methodology, project administration, writing – original draft preparation. I.B.H. data curation, formal analysis, writing – review and editing. S.D. supervision, writing – review and editing. A.T. conceptualization, data curation, formal analysis, investigation, methodology, resources, supervision, writing – review and editing.

## Ethics Statement

The study was approved by the Ethics Committee for Northwestern and Central Switzerland, approval number 2023‐00907.

## Consent

All patients have given general informed consent their archival tissue to be used for scientific research, and in accordance with the Swiss Federal Act on Research involving Human Beings, article 38, small quantities of (such archived) bodily substances form generally consented patients are allowed to be used for anonymized research purposes, as the current study.

## Conflicts of Interest

The authors declare no conflicts of interest.

## Peer Review

The peer review history for this article is available at https://www.webofscience.com/api/gateway/wos/peer-review/10.1002/hon.70145.

## Supporting information


Supporting Information S1

**Table S2:** Overview of the applied immunohistochemistry (IHC) and fluorescence in situ hybridization for *BCL2.*

**Table S3:** (A) In detail description of the detected genomic variants. (B) Explanation of the scores applied.

## Data Availability

The datasets generated during and/or analyzed during the current study are available from the corresponding author on reasonable request.
