# Supplementary material for: Tissue‐Equivalents of Lymphoid Clonal Hematopoiesis of Indeterminate Potential (L‐CHIP) and Germline‐Derived Lymphoproliferations: Possible Caveats for Hematopathologists
Source: Hematol Oncol. 2025 Oct 11;43(6):e70145. doi: 10.1002/hon.70145 (PMC12515349; doi:10.1002/hon.70145)
Supplement: Supplementary file 1 — Supporting Information S1 [file HON-43-e70145-s001.docx]

**Supplementary Material**

**Variant analysis with next generation sequencing**

H&E- and CD20-stained sections of formalin-fixed paraffin-embedded (FFPE) blocks were reviewed by A.T. to identify tissues or tissue parts with >70% B-cell content and perinodal soft tissue devoid of B-cells to be further utilized. B-cell devoid perinodal soft tissue served as a source of DNA for verification/falsification of germline variants. Depending on the distribution of cells, 25μm-thick sections were cut, or punches were taken from the tumour-rich respective tissue areas. Paraffin sections were deparaffinized and rehydrated by serial xylene and ethanol washes, respectively, and the extraction of genomic DNA was performed according to standard procedures with the Maxwell RSC FFPE Plus DNA Kit (Promega, Madison, Wisconsin, USA). DNA was treated with uracil-DNA-glycosylase (UDG, Thermo Fisher Scientific, USA) prior to the next generation sequencing (NGS) library preparation to cleave deaminated cytosines (uracils), reducing C→T or G→A mismatches that lead to common artifacts in FFPE material. DNA was quantified with the Qubit dsDNA HS Assay Kit (Molecular Probes, Thermo Fisher Scientific, USA) on the Qubit Fluorometer 2.0 (Thermo Fisher Scientific, USA). A custom-designed and later-on ISO15189 accredited IonTorrent AmpliSeq NGS lymphoma panel (Thermo Fisher Scientific, Carlsbad, CA, USA), that targets 68 genes (Suppl. Figure 1), has been used in this study (Juskevicius et al., 2016; Juskevicius et al. 2017; Pillonel et al. 2020). 40ng of DNA was used as input for library preparation whenever possible. Library preparation was carried out according to the manufacturer’s protocol for four pool panels (Thermo Fisher Scientific, USA). FFPE libraries were diluted to 50pM, loaded on Ion 550 chips by the IonChef instrument (Thermo Fisher Scientific, USA), and then sequenced on the S5 Prime sequencer (Thermo Fisher Scientific, Torrent Suite v5.16.1)

**All exons (n=20)**


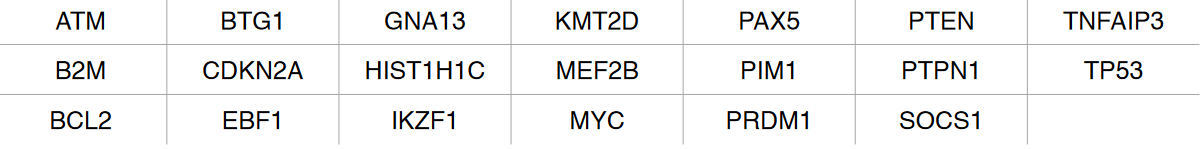


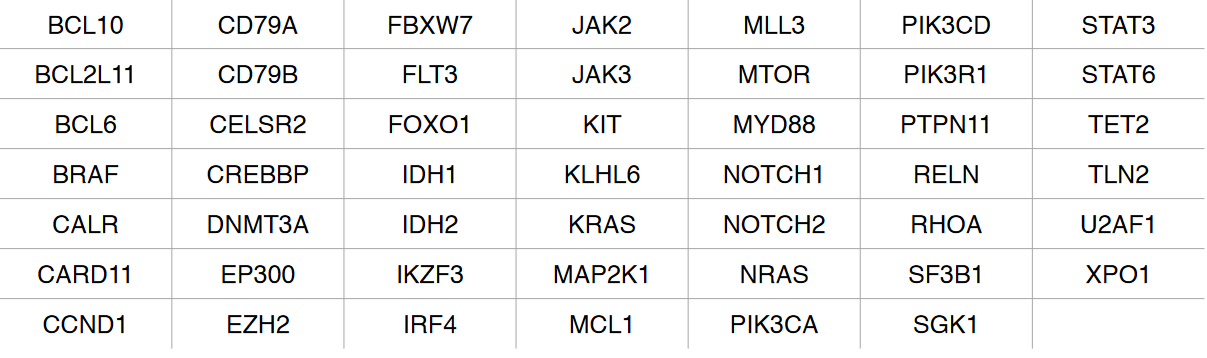
 **Hotspots only (n=48)**

| **Suppl. Figure 1:** NGS panel design |
| --- |

| The achieved mean base coverage depth was 5017× (range 1819–10547×). Raw data were processed on the Ion Torrent Server v5.16.1 and aligned to the reference genome hg19 using the Torrent Alignment Software. Only the libraries that passed quality control (>95% on target reads, <90% uniformity, and >1000x average base coverage depth) were used for further analysis. Variants were annotated with the Single Sample Annotation Workflow of the Ion Reporter (Thermo Fisher Scientific, v5.10) and dbSNP database v150 and filtered according to criteria listed in Suppl. Table 1. Detected variants were manually reviewed by the help of IGV viewer to exclude sequencing artifacts. After quality filtering, results obtained from the extracts from the B-cell enriched and the lymphocyte-devoid areas were compared to judge whether variant calls were somatic (present only in the former) or germline (present in both). For variant classification, the total number of entries of a particular amino acid change at a position in COSMIC database was considered, along with their description in at least one genomic database (ClinVar, OncoKb, VarSome). Variants were further evaluated following recommendations of the Clinical Genome Resource (ClinGen), Cancer Genomics Consortium (CGC), and Variant Interpretation for Cancer Consortium (VICC) (Horak et al., 2022), and as per ISO15189 accredited standard operation procedure of our institute. | | |
| --- | --- | --- |
| **Quality:** | **Threshold value** |  |
| Phred-based quality | >30 |  |
| Strand bias | ≤0.75 |  |
| Number of reads supporting called variant | ≥100 |  |
| **Functional relevance:** |  |  |
| Variant allelic frequency | ≥5% |  |
| Localization | Exonic and splice site |  |
| Variant effect | Non-synonymous |  |
| **SNP exclusion:** |  |  |
| Variant allelic frequency | <95% |  |
| Database annotation and alternative allelic frequency (1000 genomes project European descendent samples, ExAC non-Finnish European population) | Minor allele frequency (MAF) ≤0.01%. Not listed in dbSNP v150 or listed, but MAF ≤0.01% |  |

**Suppl. Table 1:** Criteria used for mutation filtering in targeted NGS data (variant inclusion)

**B-cell clonality analysis**

The Oncomine BCR Pan-Clonality Assay (Thermo Fisher, USA) was used to assess the B-cell clonality and diversity by analysis of the relative proportions of various B-cell heavy chains (IGH) and light chains - kappa (IGK) and Lambda (IGL) variant sequences. 200ng of DNA was used as input for library preparation whenever possible. Prepared libraries were diluted to 50pM, loaded on Ion 550 chips by the IonChef instrument (Thermo Fisher Scientific, USA), and sequenced on the S5 Prime sequencer (Thermo Fisher Scientific, Torrent Suite v5.16.1) following the manufacturer's instructions. Data were analysed by Ion Reporter Software (v5.18). A dominant rearrangement was defined if the associated sequences were more than 2.5% of the total reads and more than 10 times the polyclonal background (Arcila et al., 2019). In cases with fewer amount of tissue, B-cell clonality was assessed analysing IGH rearrangements by multiplex PCR and fragment length comparison as previously described (Meier et al., 2001).

References:

Juskevicius D, Lorber T, Gsponer J, et al. Distinct genetic evolution patterns of relapsing diffuse large B-cell lymphoma revealed by genome-wide copy number aberration and targeted sequencing analysis. Leukemia. 2016 Dec;30(12):2385-2395. doi: 10.1038/leu.2016.135. PMID: 27198204.

Juskevicius D, Jucker D, Klingbiel D, et al. Mutations of CREBBP and SOCS1 are independent prognostic factors in diffuse large B cell lymphoma: mutational analysis of the SAKK 38/07 prospective clinical trial cohort. J Hematol Oncol. 2017 Mar 17;10(1):70. doi: 10.1186/s13045-017-0438-7. PMID: 28302137.

Pillonel V, Juskevicius D, Bihl M, et al. Routine next generation sequencing of lymphoid malignancies: clinical utility and challenges from a 3-Year practical experience. Leuk Lymphoma. 2020 Nov;61(11):2568-2583. doi: 10.1080/10428194.2020.1786560. PMID: 32623938.

Horak P, Griffith M, Danos AM, et al. Standards for the classification of pathogenicity of somatic variants in cancer (oncogenicity): Joint recommendations of Clinical Genome Resource (ClinGen), Cancer Genomics Consortium (CGC), and Variant Interpretation for Cancer Consortium (VICC). Genet Med. 2022 Sep;24(9):1991. doi: 10.1016/j.gim.2022.07.001. Erratum for: Genet Med. 2022 May;24(5):986-998. PMID: 36063163.

Arcila ME, Yu W, Syed M, et al. Establishment of Immunoglobulin Heavy (IGH) Chain Clonality Testing by Next-Generation Sequencing for Routine Characterization of B-Cell and Plasma Cell Neoplasms. J Mol Diagn. 2019 Mar;21(2):330-342. doi: 10.1016/j.jmoldx.2018.10.008. PMID: 30590126.

Meier VS, Rufle A, Gudat F. Simultaneous evaluation of T- and B-cell clonality, t(11;14) and t(14;18), in a single reaction by a four-color multiplex polymerase chain reaction assay and automated high-resolution fragment analysis: a method for the rapid molecular diagnosis of lymphoproliferative disorders applicable to fresh frozen and formalin-fixed, paraffin-embedded tissues, blood, and bone marrow aspirates. Am J Pathol. 2001 Dec;159(6):2031-2043. doi: 10.1016/S0002-9440(10)63055-6. PMID: 11733354.
