# Supplementary material for: Tissue‐Equivalents of Lymphoid Clonal Hematopoiesis of Indeterminate Potential (L‐CHIP) and Germline‐Derived Lymphoproliferations: Possible Caveats for Hematopathologists
Source: Hematol Oncol. 2025 Oct 11;43(6):e70145. doi: 10.1002/hon.70145 (PMC12515349; doi:10.1002/hon.70145)
Supplement: Supplementary file 2 — Table S2: Overview of the applied immunohistochemistry (IHC) and fluorescence in situ hybridization for BCL2. [file HON-43-e70145-s003.docx]

**Supplementary Table 2.** Overview of the applied immunohistochemistry (IHC) and fluorescence *in-situ* hybridization for *BCL2*

| Case ID, | IHC phenotype | FISH (*BCL2* break apart) |
| --- | --- | --- |
| 1 | **BCL2** (SP66): negative in GC  **H3K27m3:** overexpression in GC  **PTEN:** unremarkable in GC  ARID1A and EZH2: unremarkable in GC  CD3 and CD20: unremarkable distribution  CD123: increased mature plasmacytoid dendritic cells | No *BCL2* rearrangement |
| 2 | **BCL2** (SP66, E17, and D124): negative in GC  **H3K27m3:** overexpression in GC  **PTEN:** loss of expression in GC  ARID1A and p53: unremarkable  CD21 and CD23: distorted follicular dendritic meshwork  CD79a: unremarkable distribution  EZH2: strong, unpolarized expression in GC  GCET and LMO2: physiologic expression in GC  Kappa-Lambda: polytypic plasma cells  Ki67: irregular distribution in GC  IHC for basic B- and T-cell markers were performed at an external institution and were available during consultation | No *BCL2* rearrangement |
| 3 | **H3K27m3** and **PTEN:** unremarkable in GC  ARID1A and p53: unremarkable in GC  CD21: retained follicular dendritic meshwork  IgD: retained mantle zones  Kappa-Lambda: polytypic plasma cells  Ki67: physiologic polarized distribution in GC  MUM1: only focal expression in GC; no *IRF4* rearrangement  IHC for the following markers was performed at an external institution and was available during consultation:  BCL2, BCL6, CD5, CD10, CD20, CD23, CD30, EBV (EBER ISH), PAX5 | Not performed |
| 4 | **BCL2** (SP66, E17, and D124): negative in GC  **H3K27m3** and **PTEN:** unremarkable in GC  CD5 and CD20: unremarkable distribution  CD10: negative in GC  GCET, MEF2B and stathmin: physiologic expression in GC  IgD: retained mantle zones  Ki67: irregular distribution in GC  Kappa-Lambda: polytypic plasma cells | Not performed |
| 5 | **PTEN:** loss of expression in GC | Not performed |
| 6 | **PTEN:** loss of expression in GC  CD5 and CD20: unremarkable distribution  CK20: negative | Not performed |
| 7 | **BCL2** (SP66, E17, and D124): negative in GC  **H3K27m3** and **PTEN:** unremarkable in GC  ARID1A and p53: unremarkable in GC  CD10 and MEF2B: physiologic expression in GC  CD79a: unremarkable distribution | Not performed |

**Abbreviations:** ARID1A (AT-rich interaction domain 1A), BCL2 (B-cell lymphoma 2), BCL6 (B-cell lymphoma 6), CD (cluster of differentiation), CK (cytokeratin), EZH2 (enhancer of zeste 2 polycomb repressive complex 2 subunit), EBV (ISH) (Epstein-Barr-Virus *in-situ* hybridization), GC (germinal center), GCET (germinal centre-associated lymphoma protein), H3K27m3 (tri-methylated histone 3 position lysine 27), IgD (immunoglobulin D), IHC (immunohistochemistry), LMO2 (LIM domain only 2), MEF2B (Myocyte enhancer binding factor 2B), MUM1 (multiple myeloma oncogene-1), PAX5 (paired box protein 5), PTEN (phosphatase and tensin homolog)
